# Major variations in malaria exposure of travellers in rural areas: an entomological cohort study in western Côte d'Ivoire

**DOI:** 10.1186/1475-2875-8-171

**Published:** 2009-07-28

**Authors:** Eve Orlandi-Pradines, Christophe Rogier, Bernard Koffi, Fanny Jarjaval, Melissa Bell, Vanessa Machault, Christophe Pons, Romain Girod, Jean-Paul Boutin, Frédéric Pagès

**Affiliations:** 1 Unité d'Entomologie Médicale – Unité mixte de Recherche 6236, Institut de Recherche biomédicale des Armées-antenne de Marseille, Institut de Médecine Tropicale du Service de Santé des Armées (IMTSSA), Allée du médecin colonel Eugène Jamot, Parc du Pharo, BP 60109, 13262 Marseille Cedex 07, France; 2 Unité de Recherche en Biologie et Epidémiologie Parasitaires – Unité mixte de Recherche 6236, Institut de Recherche biomédicale des Armées-antenne de Marseille, IMTSSA, 13262 Marseille Cedex 07, France; 3 CEMV Université de Bouaké, 27 BP 529, Abidjan Cedex 27, Côte d'Ivoire; 4 Service de santé du premier régiment de chasseurs parachutistes, 09100 Pamiers, France; 5 Unité d'entomologie médicale, Institut Pasteur de la Guyane, 23 avenue Pasteur, BP 6010, 97306 Cayenne Cedex, Guyane Française; 6 Département d'épidémiologie et de santé publique sud, Institut de Recherche biomédicale des Armées-antenne de Marseille, IMTSSA, 13262 Marseille Cedex 07, France

## Abstract

**Background:**

Malaria remains a major threat, to both travellers and military personnel deployed to endemic areas. The recommendations for travellers given by the World Health Organization is based on the incidence of malaria in an area and do not take the degree of exposure into account. The aim of this article is to evaluate the exposure of travellers by entomologic methods, which are the commonly used measures of the intensity of malaria transmission.

**Methods:**

From February 2004 to June 2004, five groups of 30 military personnel were stationed in up to 10 sites in western Côte d'Ivoire, from one week to several months. Adult mosquitoes were collected by human landing catches at each site during the five months and the level of exposure to malaria transmission of each group was estimated.

**Results:**

The level of transmission varied from one site to another one from less than one to approximately more than 100 infective bites per month. In the majority of sites, at least two anopheline species were involved in transmission. The cumulative EIR over the study period varied according to the groups from 29 infected bites per person/per mission to 324.

**Conclusion:**

The level of malaria transmission and malaria risk varies widely (varying by a factor of eleven) between groups of travellers travelling in the same region and at the same time. Physicians involved in travel medicine or supporting expatriated populations or refugees should consider this heterogeneity and emphasize the importance of combining appropriate measures, such as chemoprophylaxis and protective measures against mosquitoes.

## Background

Malaria remains a major threat, both for travellers and for military personnel deployed each year to endemic areas. In France in 2007, up to 4,400 cases of malaria were estimated to have been imported, including 333 cases in military personnel [1]. Chemoprophylaxis combined with anti-vector devices, such as impregnated bed nets, repellents and long-sleeve clothes, are protective measures for non-immune people travelling in malaria-endemic areas [2-6]. The growing resistance of *Plasmodium sp*. to anti-malarial drugs has led to an increasing interest in anti-vector protection measures [2]. Their efficacy in travellers has rarely been assessed and are usually only assessed by questionnaire data analysis [7]. Only one double-blinded randomized study has directly shown the efficacy of insecticide-treated clothing in reducing the incidence of malaria among travellers [8]. The main difficulty is in taking into account the exposure of the travellers to malaria transmission. Serological techniques have been used to indirectly estimate this exposure but accommodation conditions, duration and seasonality of travellers' stay in endemic areas lead to a lower exposure to malaria transmission than those of indigenous populations [9,10]. Serologic methods probably underestimate the real exposure according to the personal protection measures of individuals [11-15]. For travellers, only one remote sensing model approach has been conducted that showed a relationship between environment and malaria risk, but no quantitative risk maps could be made[16]. Rombo *et al *suggested that entomological inoculation rate (EIR) be used as a predictor of risk for non-immune persons [9]. However, the EIR that is the most commonly used measure of the intensity of malaria transmission [17] has never been estimated for travellers. It is possible to use previously published data, but reliable and timely quantitative data on area specific EIR within countries are incomplete [17,18]. In fact, travel medicine practitioners have two levels (low or high) of risk according to the type of location: urban or rural. For indigenous populations, malaria transmission in the same rural areas is very heterogeneous and malaria exposure varies between neighbouring villages or settlements [19-24]. These variations are usually not considered in the risk assessment for travellers. No studies have been conducted to determine if malaria exposure for different groups of travellers in the same rural area varies according to each group's specific itinerary in this area.

The aim of this study was to estimate the level of exposure to malaria transmission of different groups of travellers with different travel itineraries during the same period in the same rural area in western Côte d'Ivoire by entomological methods.

## Methods

### Study area

**Figure 1:**
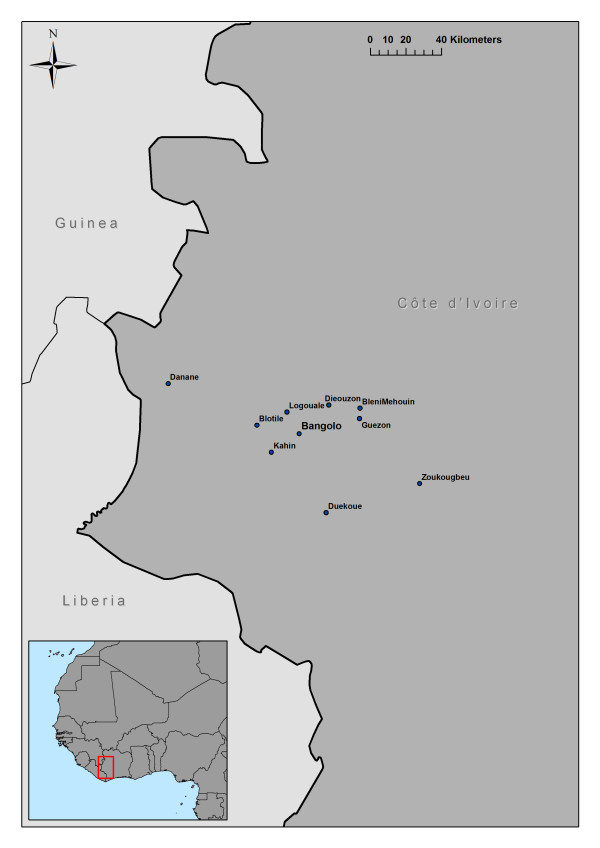
**Schematic map of Côte d'Ivoire showing the study area**.

A company of 149 French soldiers, made up of five platoons of about 30 persons, was engaged in peace-keeping operations during a five-month period (from February to June 2004) in several rural areas in Western Côte d'Ivoire (Figure 1). Western Côte d'Ivoire is considered an intense and permanent *Plasmodium falciparum *transmission area during the dry and rainy seasons [25]. The tours of duty were divided into a dry season tour, from February to March, and a rainy season tour, from April to June.

The soldiers used anti-malarial prophylaxis, including chemoprophylaxis of 100 mg of doxycycline daily, permethrin-impregnated battle dress, and deltamethrin-impregnated bed nets and repellents at night. The Army provided prophylactic drugs, anti-vectorial equipment and repellents. The military doctor for the company followed all subjects during their tour of duty and one month after their return. In the field, all fevers were recorded and Plasmodium infections were systematically investigated using rapid diagnostic test (Malaria Core ^®^, Core Diagnostics, UK). Clinical malaria attacks were defined by the association of fever and Plasmodium infection. They were reported to the military epidemiological surveillance system.

The travel itinerary of each group was recorded throughout the study and the sites where the soldiers stayed during the nights where documented with their geographical positions. The soldiers were stationed in up to 10 sites (Figure 1), for periods ranging from one week to several months: Bangolo (07,00N, 07,49W), Bleni Mehouin (07,14N, 07,18W), Danane (07,26N, 08,15W), Dieouzon (07,15N, 07,34W), Guezon (07,08N, 07,18W), Kahin (06,91N, 07,63W), Logouale (07,12N, 07,55W), Duekoue (06,61N, 07,35W), Blotile (07,05N, 07,70W) and Zoukougbeu (06,77N, 06,88W). Troops were permanently stationed in four locations: Bangolo, Guezon, Kahin and Logouale. Other sites were considered as temporary camps. At each of the sites, the EIR was estimated weekly when a group of soldiers was present. All camps were situated less than one kilometer from indigenous houses and in close proximity to potential anopheline larval sites.

#### Bangolo

Bangolo is a small town situated about 600 km north-west of Côte d'Ivoire's main city, Abidjan. It is bordered by the A7 main road going from Douekoue to Man. The military camp in Bangolo was located in the middle of the town.

#### Logouale

This site was about 1.5 km south of the town of Logouale and is bordered by the A7 main road. Some Ivoirian families lived near the camp. A swamp pool and irrigated rice paddy bordered the camp on its north side.

#### Guezon

This camp was situated at the south entry of the little village of Guezon-Nord in the forested mountain area of Mont Peko. The camp was surrounded by vegetable farms, a cocoa plantation and a swamp pool to the south.

#### Kahin

Kahin is a little village situated on the border of a river. It is surrounded by degraded forests and plantations. The camp was at the eastern outskirts of the village near the bridge on the river. During the rainy season, the borders of the camp were mainly flooded by water and the speed of the river increased.

#### Dieouzon

This is a big village with about 2,000 inhabitants situated in a deforested area surrounded by forest and cocoa plantations. The camp was in the center of the city (occupying the primary school).

#### Zoukougbeu

This camp was situated at the eastern outskirts of Zoukougbeu village, bordered by the A6 main road going to Daloa.

#### Blotile

This is a small village surrounded by forest and cocoas plantations. The camp was situated at the northern outskirts of the village, near the entry, and was bordered by the path going north to Logouale and degraded forest.

#### Bleni Mehouin

This is a big village on the northern side of Mont Peko that is surrounded by degraded forest and cocoa plantations. The camp was situated at the Western outskirts of the village and was bordered by a little stream, houses, and the path going to Dieouzon.

#### Danane

The site called Danane was in fact situated two kilometers South of Danane. The camp was bordered by the A701 main road going from Danane to Bouenneu. Two villages were situated near (< 500 m) the camp.

#### Douekoue

The site called Duekoue was in fact situated about three kilometers South of the Duekoue city, in a wood factory surrounded by forest and bordered by the A7 main road going to Guiglo.

### Field mosquito processing

Adult mosquitoes were collected by human landing catches one to two nights per week at each site during the five-month study period. Sampling was carried out at four points (two indoors and two outdoors). The total person-nights of capture indoors and outdoors at each site during the mission are shown in additional file 1: HBR, CSP and EIR of *Anopheles gambiae *s.l., *Anopheles funestus s.l*. and *Anopheles nili s.l. *by sites. Locally recruited collectors were organized into teams of two for each collection point. Workers in each team were switched out every hour from 6:00 PM to 7:00 AM. Teams of collectors were rotated among the collection points on different collection nights to minimize sampling bias. Collectors gave prior informed consent and received anti-malarial prophylaxis and yellow fever immunization.

The species of mosquitoes were recorded by day, site, and hours of capture. They were sorted by Genus and anopheline mosquitoes were identified morphologically following the keys of Gillies and Coetzee [26] and using the software developed by Hervy *et al *[27]. Culicinae were identified morphologically following the Edwards keys [28]. All mosquitoes were stored individually in numbered vials with desiccant and preserved at -20°C until processing at the Medical Entomology Unit of the Institute for Tropical Medicine (IMTSSA), Marseille (France).

### Laboratory mosquito processing

#### *Plasmodium falciparum *infection

The presence of the circumsporozoite protein (CSP) of *Plasmodium falciparum *was tested for using enzyme-linked immunosorbent assay (ELISA) [29,30]. All anopheline females were tested at temporary sites. At permanent sites, all anopheline females were tested for *P. falciparum *CSP, when there were less than 100 specimens or at least a random sample of 100 specimens by species and sorted by season (rainy or dry season).

#### Molecular identification

For each site and month of capture, all the specimens were analyzed for molecular identification if they were fewer than 100. When they were more than 100, a random sample of 100 specimens was considered for the molecular analysis. Moreover, all CSP positive specimens were identified by molecular methods.

Females belonging to the *An. gambiae *complex, the *An. funestus *and *An. nili *groups were identified by polymerase chain reaction (PCR) [31-33] at the species level. *Anopheles gambiae s.s*. were molecularly distinguished into molecular forms [31]. When M/S hybrids were identified, the specimens were tested again using the same DNA extract and, if the results remained the same, a new DNA extraction was done to confirm this result as previously described [34,35]. Data on resistance are not presented in this paper.

### Data analysis

The human biting rate (HBR) was expressed as the average number of female anopheline bites per person per night during the dry season and rainy season. Specific CSP rates were calculated per species, per site and per season as the proportion of mosquitoes found to contain circumsporozoite antigen by ELISA. Ninety-five percent confidence intervals were calculated by the exact binomial method. Comparisons of these proportions were performed by Chi-square (p values < 0.05 were considered to be statistically significant). Specific entomological inoculation rates (EIR) were calculated as the product of the HBR per site, per season for *An. gambiae s.l., An. funestus s.l. *and *An. nili s.l. *and their respective CSP rates.

The malaria attack rate was calculated as the ratio of the number of clinical malaria cases to the number of soldiers participating in the mission.

## Results

### Mosquito diversity

During the five-month period, a total of 12,520 female mosquitoes were caught during 81 collections nights (195 person-nights of capture) at ten different localities: 51.4% were *Anopheles*, 20.8% were *Culex*, and 2.3% were *Aedes*, mainly *Aedes aegypti *(41.8%). In Logouale, the number of catching points was reduced to two (one indoors and one outdoors) after the first collection night due to the high number of *Anopheles sp *caught in this place. Anopheles were caught indoors (48% of captured specimens) as well as outdoors (52%). Of the 6,435 female anopheles collected, 65.2% were *An. gambiae s.l.*, 19% *An. funestus s.l. *and 13.7% *An. nili s.l*. They were found at all sites, except in Zoukougbeu and Douekoue where *An. nili *was not caught. Other anopheline species were captured, including *Anopheles pharoensis *(mainly in Logouale), *Anopheles hancocki *(mainly in Guezon), *Anopheles dureni *and *Anopheles ziemanni*.

Out of the 4,197 *An. gambiae s.l. *collected, 1,626 were identified by PCR. They were all *An. gambiae s.s. *with *An. gambiae s.s*. M form (48.2%), M/S form (0.3%) and S form (51.5%). Out of the 1,219 *An. funestus s.l. *collected, 695 were identified by PCR, 99.1% were *An. funestus s.s *and 0.9% *Anopheles leesoni *(three specimens in Danane and one each in Logouale, Guezon and Bleni Mehouin). Out of the 880 *An. nili s.l. *collected, 396 were identified by PCR. They were all *An. nili s.s.*

### Molecular identification of *An. gambiae s.s*

**Figure 2:**
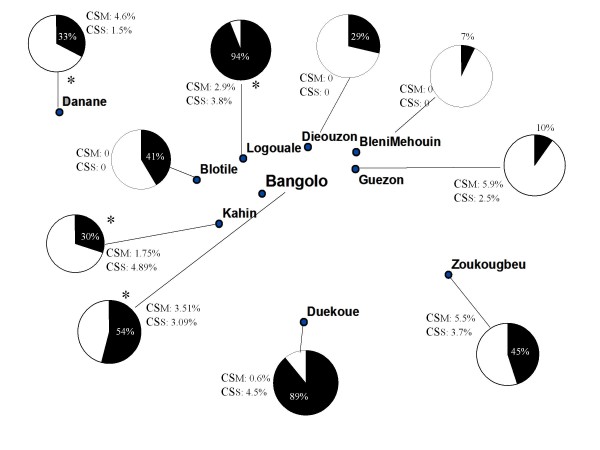
**Distribution of *Anopheles gambiae *s.s. molecular forms populations and respective CSP rates**. Sample percentage of the M and S molecular forms in each population are indicated in black and white respectively. * represents the presence of one hybrid M/S CSM: CSP rates of M molecular form *An. gambiae s.s*. CSS: CSP rates of S molecular form *An. gambiae s.s*.

M and S molecular forms cohabitated all sites and their distribution is presented in Figure 2. Of the *An. gambiae s.s*. captured at each permanent site, more than 90% in Bangolo and Guezon, 68% in Kahin, and 15% in Logouale were identified. *Anopheles gambiae s.s.*, identified as the S molecular form, was most common in Guezon and Kahin, while the M molecular form was most common in Logouale (Figure 2). In Bangolo, the two forms were found in the same proportions (Figure 2). Four hybrids M/S form were identified at four sites (Figure 2). There was no difference in molecular forms between the dry and rainy seasons at all permanent sites.

### Entomological parameters, seasonality and spatial variation

The entomological data on temporary sites and permanent sites are summarized in additional file 1: HBR, CSP and EIR of *An. gambiae *s.l., *An. funestus s.l*. and *An. nili s.l. *by sites. Only the data obtained at permanent sites are presented below (Table 1).

**Table 1:** Mean daily human biting rate (HBR), circumsporozoite protein rates (CSP) and entomological inoculation rates (EIR – infective bites per person per months) of *An. gambiae *s.l., *An. funestus s.l*. and *An. nili s.l. *by permanent sites.

		Dry period				Rainy period			
Permanent Sites		*An. gambiae s.l.*	*An. funestus s.l.*	*An. nili s.l.*	all vectors	*An. gambiae s.l.*	*An. funestus s.l.*	*An. nili s.l.*	all vectors
Bangolo	PNC: 13/28								

	Total collected	7	0	0	7	265	38	3	306
									
	HBR	0.5	-	-	0.5	9.5	1.4	0.1	10.9
	Tested for CSP	7	-	-	7	258	37	3	298
	CSP rate [95% C.I.]	0% [0–42.0]	-	-	0% [0–42.0]	2.7% [1.1–5.5]	2.7% [0.1–14.2]	0% [0–70.8]	2.7% [1.2–5.2]
	EIR	0	-	-	0	7.8	1.1	0	8.9
									
Guezon	PNC: 20/28								
	Total collected	30	122	0	152	162	348	2	512
									
	HBR	1.5	6.1	-	7.6	5.8	12.4	0.1	18.3
	Tested for CSP	30	95	-	117	161	346	2	509
	CSP rate [95% C.I.]	0% [0–15.4]	2.1% [0.3–7.4]	-	1.7% [0.2–6.0]	3.7% [1.4–7.9]	5.5% [3.3–8.4]	0% [0–84.2]	4.9% [3.2–7.2]
	EIR	0	3.9	-	3.9	6.6	20.7	0	27.3
									
**Kahin**	PNC: 7/23								
	Total collected	185	44	394	623	375	131	386	892
									
	HBR	26.4	6.3	56.3	89	16.3	5.7	16.8	38.8
	Tested for CSP	100	44	197	293	306	117	303	726
	CSP rate [95% C.I.]	3.0% [0.3–9.0]	11.4% [3.8–24.6]	3.5% [1.4–7.2]	3.7% [1.9–6.6]	4.2% [2.3–7.2]	2.6% [0.5–7.3]	1.0% [0.2–2.9]	2.6% [1.6–4.1]
	EIR	23.8	21.5	60.8	102.7	21.1	4.4	5.1	30.6
									
**Logouale**	PNC: 6/12								
	Total collected	565	10	23	598	1531	43	34	1608
									
	HBR	94.2	1.7	3.8	99.7	127.6	3.6	2.8	134
	Tested for CSP	99	10	23	132	749	40	34	823
	CSP rate [95% C.I.]	3% [0.6–8.6]	0% [0–30.9]	0% [0–14.8]	2.3% [0.5–6.5]	2.9% [1.9–4.4]	0% [0–8.8]	0% [0–10.3]	2.7% [1.7–4]
	EIR	86.0	0	0	86.0	114.1	0	0	114.1

The dry period goes from February to March 2004 and the rainy period goes from April to June 2004.

HBR: Mean daily human biting rate

CSP: circumsporozoite protein rates

EIR: entomological inoculation rates – infective bites per person per month

PNC: Total Person-night of capture in dry season/rainy season

95% C.I.: 95% confidence interval

#### HBR and spatial variation

The mean daily HBR varied from 0.5 to 99.7 and from 10.9 to 134, during the dry and the rainy seasons, respectively. The highest HBR was always observed at Logouale. The mean daily HBR increased with rainfall at all the sites except Kahin where HBR decreased from 89 to 38.8 bites/person/night (Table 1). *Anopheles gambiae s.l. *was the predominant vector species in Bangolo and Logouale, *An. nili s.l*. in Kahin, and *An. funestus s.l. *in Guezon (Table 1).

#### HBR and time variation

The evolution by months and by species of the HBR is given in additional file 2 for the four permanent sites.

#### Biting behaviour

In Bangolo, *An. gambiae *s.l and *An. funestus *s.l. were more exophagic than endophagic (52% vs. 48% and 55% vs. 45% respectively; differences not statistically significant). In Guezon, *An. gambiae *s.l. was exo-endophagic (47% vs. 53%, difference not statistically significant) and *An. funestus *s.l. was more endophagic (37% of bites occurred outdoors vs. 63% indoors. p < 0.05). In Kahin, *An. gambiae *s.l. and An. nili s.l. were exophagic and *An. funestus *s.l. was exo-endophagic. For *An. gambiae *s.l., 64% of bites occurred outdoors vs. 36% (p < 0.05). For An. nili s.l., 60% vs. 40% outdoors (p < 0.05), and for *An. funestus *s.l. 47% vs. 53% (difference not statistically significant). In Logouale, *An. gambiae *s.l. and An. nili s.l. were exo-endophagic and *An. funestus *s.l. was exophagic. For *An. gambiae *s.l., 52% of bites occurred outdoors vs. 48% (difference not statistically significant). For An. nili s.l., 42% vs. 58% (difference not statistically significant), and for *An. funestus *s.l., 75% vs. 25% (p < 0.05).

**Figure 3:**
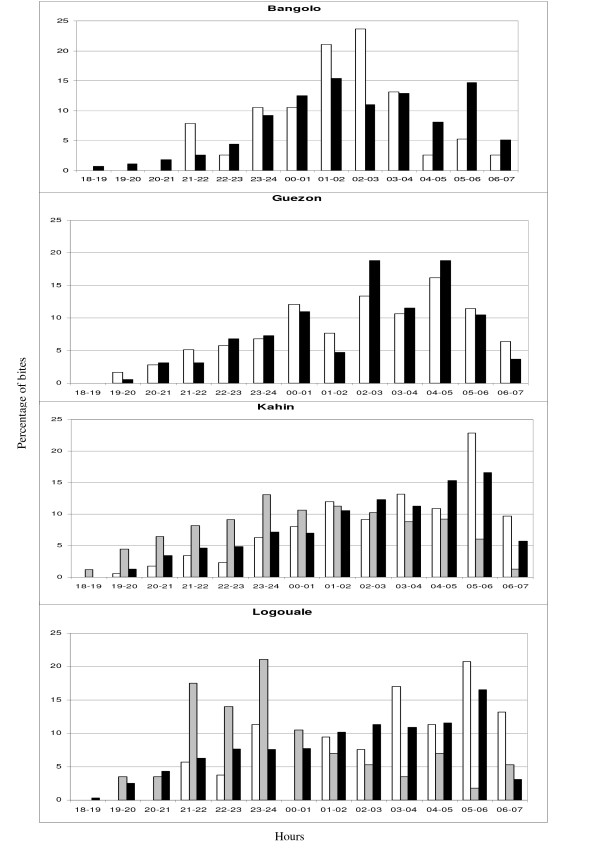
**The nocturnal biting cycle of *An. gambiae *s.l., *An. funestus s.l*. and *An. nili s.l. *by permanent sites**. Black represents the percentage of bites by hour of *An. gambiae *s.l., and white and grey bars represent the percentage of bites by hour *An. funestus s.l*. and *An. nili s.l*, respectively.

The distribution of *An. gambiae s.l., An. funestus s.l. *and *An. nili s.l*. bites per hour per site is shown in Figure 3. More than 75% of bites occurred between midnight and 6:00 a.m. at all sites and 4% were recorded between 6:00 a.m. and 7:00 a.m. The frequency of *An. gambiae s.l. *and *An. funestus s.l *bites increased gradually between 7:00 p.m. and 1:00 a.m. reaching different peaks at each site. In Bangolo, the peak frequencies of bites were between 1:00 a.m. and 3:00 a.m., in Guezon between 4:00 a.m. and 5:00 a.m., and in Logouale and Kahin between 5:00 a.m. and 6:00 a.m.

The frequency of *An. nili s.l. *bites was observed to increase gradually between 7:00 p.m. and 11:00 p.m. at all sites, and to peak between 11:00 p.m. and 00:00 a.m. and then decrease.

#### CSP rate and spatial variation

Ninety of the 2,905 (3%) anopheline vectors were found to be positive by ELISA for circumsporozoite protein detection. The results by sites and by species are shown in Table 1. *An. gambiae *s.l. populations were found to be infected at both permanent sites and their CSP rates did not differ significantly between sites (p = 0.67). *An. funestus *s.l. populations were found to be infected at all sites except Logouale and their CSP rates did not differ significantly among sites (p = 0.36). Whatever the period, An. nili s.l, was found to be infected only in Kahin with a significant decrease between the dry and rainy period (p = 0.04). The CSP index for *An. gambiae *s.l. between the dry and the rainy season did not differ significantly. For *An. funestus *s.l., the CSP rates did not differ significantly between the dry and the rainy season expect in Kahin where it decreased in the rainy season (p = 0.03).

Random samples of *An. gambiae s.l*. caught during the five months at each site were identified at the species and molecular level and were all tested for CSP. None of the three M/S hybrids was infected. The difference between the CSP rates for *An. gambiae *M and S molecular form was not significant at any site. The CSP rates of *An. gambiae *M and S forms varied between the permanent sites from 1.75% to 10% (Figure 2). These differences were significant for M form (p = 0.036), not for S form (p = 0.4). The differences between CSP rates by period were not significant at all sites (p > 0.2).

#### EIR and spatial variation

During the five-month mission, the mean monthly EIR was 8.9 infective bites in Bangolo, 31.2 in Guezon, 133.3 in Kahin and 200.1 in Logouale. During the dry period, EIR varied from 0 to 102.7 according to the site (Table 1). During the rainy season, EIR varied from 8.9 to 114.1 according to the site (Table 1). *Anopheles gambiae *s.l. was involved in *P. falciparum *transmission at all sites and was the main vector in Bangolo and Logouale. *Anopheles funestus s.l. *was the main vector in Guezon and was involved in *P. falciparum *transmission in Bangolo and Kahin. *Anopheles nili s.l. *was significantly involved in malaria transmission only in Kahin. During the dry season, it was the main vector in this area but its EIR decreased from 60.8 during the dry season to 5.1 during the rainy period (Table 1).

### Exposure of soldiers to anopheline vectors and malaria incidence

**Figure 4:**
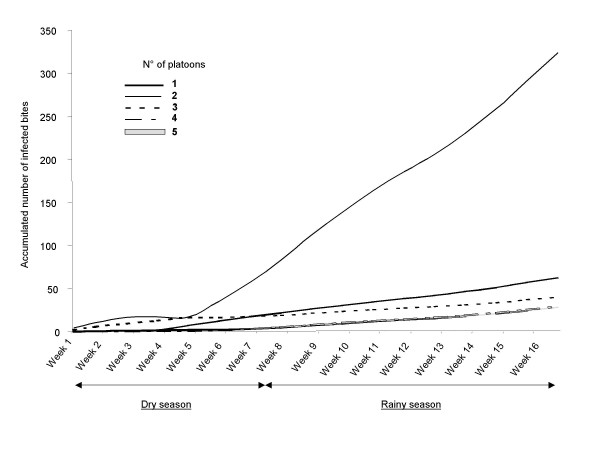
**The average accumulated number of infected anopheline bites sustained by a platoon of French military personnel**.

The number of weeks spent by each platoon at each site was presented in Table 2. The average EIR was estimated by platoon according to the EIRs estimated at each place where they stayed during the five months. The cumulative EIR over the mission varied according to the platoons (Figure 4): 324 infected bites/per person for platoon 2, 63 for platoon 1, 40 for platoon 3 and 29 for platoons 4 and 5. These cumulative EIRs increased during all the duty.

**Table 2:** Number of weeks spent by each platoon of French military company in each site in western Côte d'Ivoire during a five-month mission from February to June 2004.

Platoons	**Bangolo**	**Danané**	**Kahin**	**Logoualé**	**Guezon**	**BleniMéhouin**	**Dieouzon**	**Zoukougbeu**	**Blotile**	**Duekoue**
1			15		3					0.5
2				14			2	2	2	
3	4	4			10					
4	10				4	4				
5	15				3					

During their stay, there were six cases of malaria (three cases in platoon 4, two cases in platoons 3 and one case in platoon 2). Five cases occurred in returnees: two cases in platoon 5 and 3 and one case in platoon 1. The malaria incidence rates were of 4.1% in Côte d'Ivoire and 3.4% after returning in France (global 7.4%).

## Discussion

The findings of this study clearly show that the level of malaria transmission and malaria risk varies widely (varying by a factor of eleven) between groups of travellers travelling in the same region and at the same time. Currently, the World Health Organization [36] and travel medicine practitioners only consider travellers to rural areas to have a higher risk of exposure to disease vectors in their recommendations. They do not take into account the heterogeneity of transmission in urban or rural areas in the same district [37,38]. Differences between human individuals in their attractiveness to female mosquitoes [39], in chemoprophylaxis observance, and in compliance with the use of anti-vector devices are not taken into account for assessment of individual risk for malaria, although they are determining factors for clinical malaria in travellers.

The level of transmission varied greatly from one site to another. These results are consistent with studies conducted in other parts of Africa that showed an important heterogeneity in malaria transmission between rural areas [40-43] and within the same rural area [19-24]. In this study, EIR varied between study sites from less than one to approximately 100 infective bites per months during the dry season, and from less than nine to approximately 115 infective bites per months during the rainy season. During the duty, malaria transmission increased overall during the rainy season but was also very high in some places during the dry season like in Kahin due to the presence of *An. nili *that thrive on the border of the river. This study confirms that, in wet savannah areas or in mountainous forested areas of Côte d'Ivoire, the rainy season, which occurs for most months in the year, allows the breeding sites to retain water much longer and thus sustain more substantial residual vector populations capable of ensuring malaria transmission even during the dry season [44-46]. In all sites, CSP rates did not vary with the increase in rainfall. The variations in malaria transmission were essentially caused by the variations in HBR, probably because of variations in the availability of breeding sites. These results confirm the heterogeneity of malaria transmission in rural areas [43] and the seasonal variation in malaria in western Côte d'Ivoire [47]. Travel medicine practitioners should be very prudent in counseling travellers. Travel during the dry season is not synonymous with low transmission levels and data extracted from literature to predict the malaria risk may not be relevant in another place in the same region. HBRs and EIRs were greater than previously reported in Danane [25] and Man areas [45,46], respectively. It is difficult explain whether these difference are due to anthropic modifications of the landscape [47-49], to geographical and ecological differences at the study sites, or the greater sensitivity of ELISA CSP in *P. falciparum *detection than the salivary glands detection that was used in previous studies.

Although *An. gambiae s.s*. and *An. funestus s.s*. were individually the only significant malaria vectors at three sites (Zoukougbeu, Logouale and Dieouzon), in the other sites, the transmission was carried out by a vector association of at least two anopheline species, as previously described in Côte d'Ivoire [44,50-52]. The main vectors were principally *An. gambiae *s.s. and *An. funestus *s.s,. with *An. nili s.s*. in a few places. In Guezon, *An. funestus s.s. *was the main vector during both dry and rainy seasons, and *An. gambiae s.s *was involved in transmission only during the rainy season. In Bangolo, *An. gambiae s.s. *was only found infected during the rainy season. The few human landing collections conducted here during the dry season did not allow *P. falciparum*-infected *specimens *to be caught, but *An. gambiae s.s. *was probably involved in malaria transmission in the dry season too. On the other hand, only a few specimens of *An. funestus s.s*., including one infected specimen, were caught in Bangolo, and these were only caught during the rainy season. *Anopheles funestus s.s*. was also only involved in malaria transmission as a secondary vector during the rainy season. In Logouale, *An. gambiae *s.s., *An. funestus *s.s. and *An. nili s.s*. were present during the dry and rainy seasons, but only *An gambiae s.s. *seemed significantly involved in malaria transmission. Indeed, the low *An. funestus s.s. *and *An. nili s.s *HBRs did not allow them to play a significant role in malaria transmission. In Kahin, *An. gambiae *s.s., *An. funestus *s.s. and *An. nili s.s*. were involved in malaria transmission during the dry and rainy seasons. *Anopheles gambiae s.s *was the main vector during the rainy season. *Anopheles nili s.s *was the main vector during the dry season. During this period, the EIR was above 60 infective bites by *An. nili s.s. Anopheles nili *breeding sites were probably located on the borders of the local river. During the wet season, the increase in the river water level probably destroyed most *An. nili *larval habitats. This is probably the explanation for the *An. nili *EIR and overall EIR decrease in Kahin during the rainy season. Others species were caught such as *An. leesoni, An. hancocki, An. pharoensis *and *An. ziemanni*, but none of them were found to be infected. During the study, in most of sites, the three dominant vectors bit humans as often indoors as they did outdoors.

Most of *An. nili s.l. *bites occurred in the first part of the night. In Kahin, four of the ten infected *An. nili *were caught between 9.00 p.m. and midnight These findings (exophagic behaviour, biting cycles) could limit the impact of insecticide-treated mosquito nets for local populations or travellers when they are exposed to *An. nili *bites. Furthermore, this study showed that 4% of bites occurred between 6:00 a.m. and 7:00 a.m. At the four permanent sites, three of the 27 infected *An. funestus *and 10 of the 53 infected *An. gambiae *were caught 6:00 a.m. and 7:00 a.m. Consequently, measures for anti-vector protection used in French military from 6:00 p.m. to 6:00 a.m., such as the wearing of long-sleeve clothes or the use of repellents, have been prolonged till 7:00 a.m. daily. These results showed that malaria transmission also occurs in the first hours of the day. Entomological studies often stopped the captures on 6:00 a.m., and, in some parts of Africa, they are probably underestimating the level of transmission.

The M form was predominant in sites where vegetation cover was deeper, like in Logouale and Douekoue. The S form was predominant in mountainous areas where forest cover was highly degraded (Guezon and Bleni Mehouin). In other places, the S-part of the population increased with anthropic modifications of the environment. This result is consistent with data from Wondji *et al *in Cameroon [53]. The variations in proportions of M and S forms between sites could be explained by different breeding conditions: The M-form is considered to be more adapted to colonize semi-permanent larval sites, while the S-form is more adapted to rain-dependent temporary breeding sites [54]. Interestingly, there was no difference in *An. gambiae s.s*. population composition between the dry and rainy seasons, as described by Della Torre *et al *in Burkina Faso [55]. This discrepancy could perhaps be attributed to a different repartition in chromosomal forms of *An. gambiae s.s*. between western Côte d'Ivoire and Burkina Faso.

Although exposure to malaria transmission is high, very few malaria cases occurred among the soldiers. Eleven cases were recorded: 54.5% of which occurred in Côte d'Ivoire and 45.5% after returning to France. These proportions are similar to those observed for the French Army from 1998 to 2006 [56]. The attack rates by platoon were not associated with the level of exposure and most of cases occurred in the less exposed platoons (platoons 3, 4 and 5). This result could be explained by the high anopheline aggressiveness in some places, as was the case in Logouale. Soldiers were very interested in this study and in human landing catches. They saw the results of the catches each night. In some places, they could have increased their compliance to the use of personal protection devices against vectors and chemoprophylaxis. This bias could explain the lower incidence of malaria in platoon 2. The impact of an increase in protective behaviors and their effects on malaria exposure has already been described in indigenous populations exposed to high anopheline aggressiveness when compared to less exposed populations in the same areas in Mali, Tanzania or Côte d'Ivoire [48,57,58]. Other studies have shown the importance of personal protection measures in decreasing malaria infection risk in soldiers [8,59]. This study also suggests the important role of preventive measures and personal protection in mitigating the risk of disease. In areas of high transmission, these measures seem highly effective, as was observed in platoon 2. The improvement of the efficacy of the malaria prevention policy needs probably to improve the compliance with the preventive measures and the identification of the localities at higher risk of transmission in order to avoid the lasting deployment of troops in these settings.

## Conclusion

Malaria transmission in rural area is highly heterogeneous. The malaria exposure of travellers is highly dependent on this heterogeneity. Physicians involved in travel medicine or supporting expatriated population or refugees should emphasize the importance of vector control measures in protecting these populations (expatriated population or refugees) or counsel travellers before their stay in malaria transmission area. In addition, anti-vectorial measures are actually the only recommended measures for protection against vectors that transmit other pathogens, such as arboviruses.

## Competing interests

The authors declare that they have no competing interests.

## Authors' contributions

EOP: Contributed substantially to conception, study design, data collection, analysis and interpretation, preparation of the manuscript CR: conceived of the study, and participated in its design and coordination, data collection and critically reading the manuscript for important intellectual content. BK and CP: carried out data collection and trained the field staff. FJ, RG, VM and MB: processed entomological specimens in the laboratory, compiled the data and carried out statistical analyses. JPB: Participation in study design, coordination and critically reading of the manuscript. FP: Contributed substantially to conception, study design, data collection, analysis and interpretation, performed the statistical analysis and helped to draft the manuscript. All authors read and approved the final manuscript.

## Financial support

This study was supported by Délégation Générale pour l'Armement (grant 02CO011, no. 010808) with the valuable assistance of the veterinary Chief Claire Dane.

## Supplementary Material

Additional file 1 **By site, HBR, CSP and monthly EIR of *An. gambiae s.l*., *An. funestus s.l. *and *An. nili s.l. *and number of species identified by molecular methods**. The data provided in a table represent the results of human landing catches at each permanent and temporary sites realized from February to June 2004. The total Person-night of capture in dry season/rainy season (PNC), mean daily human biting rates (HBR), circumsporozoite protein rates (CSP) and entomological inoculation rates infective bites per person per month (EIR) of *An. gambiae s.l*., *An. funestus s.l. *and *An. nili s.l. *in each site are shown. The species identified by molecular methods and their number found in each site are presented too. 95% C.I.: 95% confidence interval. Click here for file

Additional file 2 **By permanent site, monthly HBR of *An. gambiae s.l.*, *An. funestus s.l. *and *An. nili s.l. *from February to June 2004**. The data provided represent the mean daily human biting rate (HBR) of *An. gambiae s.l.*, *An. funestus s.l. *and *An. nili s.l. *calculated by month, from February to June 2004, in four permanent sites. PNC: Total Person-night of capture. Click here for file
