# Supplementary material for: Major variations in malaria exposure of travellers in rural areas: an entomological cohort study in western Côte d'Ivoire
Source: Malar J. 2009 Jul 28;8:171. doi: 10.1186/1475-2875-8-171 (PMC2726180; doi:10.1186/1475-2875-8-171)
Supplement: Additional file 1 — By site, HBR, CSP and monthly EIR of An. gambiae s.l., An. funestus s.l. and An. nili s.l. and number of species identified by molecular methods. The data provided in a table represent the results of human landing catches at each permanent and temporary sites realized from February to June 2004. The total Person-night of capture in dry season/rainy season (PNC), mean daily human biting rates (HBR), circumsporozoite protein rates (CSP) and entomological inoculation rates infective bites per person per month (EIR) of An. gambiae s.l., An. funestus s.l. and An. nili s.l. in each site are shown. The species identified by molecular methods and their number found in each site are presented too. 95% C.I.: 95% confidence interval. [file 1475-2875-8-171-S1.doc]

|  | **Temporary sites** | | | | | | **Permanent sites** | | | |
| --- | --- | --- | --- | --- | --- | --- | --- | --- | --- | --- |
| Sites | **Danane** PNC : 16 | **BleniMehouin**  **PNC : 16** | **Dieouzon**  **PNC : 10** | **Zoukougbeu**  **PNC : 8** | **Douekoue**  **PNC : 4** | **Blotile**  **PNC : 4** | **Bangolo**  **PNC : 41** | **Guezon**  **PNC : 48** | **Kahin**  **PNC : 30** | **Logouale**  **PNC : 18** |
| ***An. gambiae s.l.*** |  |  |  |  |  |  |  |  |  |  |
| Total collected | 195 | 14 | 138 | 244 | 399 | 29 | 272 | 192 | 560 | 2096 |
| HBR | 12.2 | 0.9 | 13.8 | 30.5 | 99.8 | 7.3 | 6.6 | 4 | 18.7 | 116.4 |
| Tested for CSP | 195 | 14 | 138 | 244 | 399 | 29 | 265 | 183 | 384 | 848 |
| CSP rate  [95% C.I.] | 2.6%  [0.8-5.9] | 0%  [0-23.2] | 0%  [0-2.6] | 4.5%  [2.3-7.9] | 1.2%  [0.4-2.9] | 0%  [0-11.9] | 2.6%  [1.1-5.4] | 3.3%  [1.2-7.0] | 3.9%  [2.2-6.4] | 2.9%  [1.9-4.3] |
| EIR | **9.5** | **0** | **0** | **41.8** | **37.9** | **0** | **5.3** | **4.0** | **22.2** | **104.4** |
| *An. gambiae M form*  *An. gambiae M/S form*  *An. gambiae S form* | 34  1  70 | 1  13 | 39  97 | 50  61 | 74  9 | 12  17 | 114  1  97 | 17  158 | 114  1  266 | 301  1  20 |
|  |  |  |  |  |  |  |  |  |  |  |
| ***An. funestus s.l.*** |  |  |  |  |  |  |  |  |  |  |
| Total collected | 227 | 46 | 134 | 1 | 63 | 14 | 38 | 470 | 175 | 53 |
| HBR | 14.2 | 2.9 | 13.4 | 0.1 | 15.8 | 3.5 | 0.93 | 9.8 | 5.8 | 2.9 |
| Tested for CSP | 226 | 46 | 132 | 1 | 62 | 11 | 37 | 441 | 135 | 50 |
| CSP rate  [95% C.I.] | 4.9%  [2.5-8.5] | 10.9%  [3.6-23.6] | 2.3%  [0.5-6.5] | 0%  [0-97.5] | 1.6%  [0 –8.7] | 9.1%  [0.2-41.3] | 2.7%  [0.1-14.2] | 4.8%  [3.0-7.2] | 3.7%  [1.2-8.4] | 0%  [0-7.1] |
| **EIR** | **21.0** | **9.6** | **9.3** | **0** | **7.7** | **9.7** | **0.8** | **14.2** | **6.5** | **0** |
| *An. funestus s.s.*  *An. leesoni* | 135  3 | 31  1 | 105 | 1 | 33 | 13 | 33 | 210  1 | 85 | 41  1 |
|  |  |  |  |  |  |  |  |  |  |  |
| ***An. nili s.l.*** |  |  |  |  |  |  |  |  |  |  |
| Total collected | 26 | 3 | 5 | 0 | 0 | 4 | 3 | 2 | 750 | 57 |
| HBR | 1.6 | 0.2 | 0.5 |  |  | 1 | 0.07 | 0.04 | 25 | 3.2 |
| Tested for CSP | 26 | 3 | 5 |  |  | 4 | 3 | 2 | 500 | 57 |
| CSP rate  [95% C.I.] | 3.8%  [0.1-19.6] | 0%  [0-70.8] | 0%  [0-52.2] |  |  | 0%  [0-60.2] | 0%  [0-70.8] | 0%  [0-84.2] | 2%  [1-3.6] | 0%  [0-6.3] |
| **EIR** | **1.9** | **0** | **0** |  |  | **0** | **0** | **0** | **15.2** | **0** |
| *An. nili.s.s.* | 26 | 3 | 5 |  |  | 4 | 2 | 2 | 299 | 55 |

Additional file 1: Mean daily human biting rate (HBR), circumsporozoite protein rates (CSP) and entomological inoculation rates (EIR – infective bites per person per month) of *An. gambiae* s.l., *An. funestus s.l*. and *An. nili s.l.* by sites in western Côte d’Ivoire from February to June 2004.
