# Supplementary material for: Major variations in malaria exposure of travellers in rural areas: an entomological cohort study in western Côte d'Ivoire
Source: Malar J. 2009 Jul 28;8:171. doi: 10.1186/1475-2875-8-171 (PMC2726180; doi:10.1186/1475-2875-8-171)
Supplement: Additional file 2 — By permanent site, monthly HBR of An. gambiae s.l., An. funestus s.l. and An. nili s.l. from February to June 2004. The data provided represent the mean daily human biting rate (HBR) of An. gambiae s.l., An. funestus s.l. and An. nili s.l. calculated by month, from February to June 2004, in four permanent sites. PNC: Total Person-night of capture. [file 1475-2875-8-171-S2.doc]

| Permanent sites |  | February -March | April | May | June |
| --- | --- | --- | --- | --- | --- |
| Bangolo | PNC | 13 | 12 | 12 | 4 |
|  | An. gambiae | 0.5 | 11.3 | 4.8 | 17.8 |
|  | *An. funestus* | 0 | 0.7 | 0.9 | 4.75 |
|  | *An. nili* | 0 | 0.1 | 0.1 | 0.3 |
|  |  |  |  |  |  |
| Guezon | PNC | 20 | 16 | 8 | 4 |
|  | An. gambiae | 1.5 | 6.5 | 2 | 10.5 |
|  | *An. funestus* | 6.1 | 12.9 | 9.5 | 16.3 |
|  | *An. nili* | 0 | 0.1 | 0.1 | 0 |
|  |  |  |  |  |  |
| Kahin | PNC | 7 | 9 | 11 | 3 |
|  | An. gambiae | 26.4 | 18.8 | 9.7 | 33 |
|  | *An. funestus* | 6.3 | 2.9 | 6.7 | 10.3 |
|  | *An. nili* | 56.3 | 15.3 | 14.9 | 28 |
|  |  |  |  |  |  |
| Logouale | PNC | 6 | 6 | 4 | 2 |
|  | An. gambiae | 94.2 | 151.7 | 74.3 | 162 |
|  | *An. funestus* | 1.7 | 3 | 3.75 | 5 |
|  | *An. nili* | 3.8 | 3.8 | 1.25 | 3 |

**Additional file 2**: **By permanent site, monthly HBR of *An. gambiae s.l.,* *An. funestus s.l*. and *An. nili s.l.* from February to june 2004.**

HBR: Mean daily human biting rate

PNC: Total Person-night of capture in dry season/ rainy season
